# CLINICAL-EPIDEMIOLOGICAL PROFILE OF 106 PEDIATRIC PACIENTS WITH UROLITHIASIS IN RIO DE JANEIRO, BRAZIL

**DOI:** 10.1590/1984-0462/;2018;36;3;00009

**Published:** 2018

**Authors:** Clarisse Barbosa Barata, Cristina Ortiz Sobrinho Valete

**Affiliations:** a Universidade Federal Fluminense, Niterói, RJ, Brasil.

**Keywords:** Pediatrics, Urolithiasis, Health profile, Pediatria, Urolitíase, Perfil de saúde

## Abstract

**Objective:**

To describe the frequency, clinical profile and treatment of patients with urolithiasis in the Pediatric Nephrology Department of a public state hospital in Rio de Janeiro, Brazil.

**Methods:**

Retrospective study. Data from pediatric patients (age: 1 month - 18 years) with urolithiasis admitted between January/2012 and December/2014 were reviewed from hospital charts. The studied variables were: demographic and anthropometric data, clinical status, family history of urolithiasis, urinary tract infection and use of lithogenic drugs, diagnostic procedures, associated abnormalities, metabolic disorders, treatment and recurrence.

**Results:**

The frequency of urolithiasis was 13.6%. Main characteristics of the patients: male gender, white race, eutrophy, aged between 5 and 10 years, family history of urolithiasis, previous urinary infection and spontaneous stone passage. Abdominal and flank pain and macroscopic hematuria were the most common complaints. The most frequent metabolic disorders were hypercalciuria, hyperuricosuria and hypocitraturia. Hypocitraturia was associated with previous urinary infection (p=0.004). Abdomen/urinary tract ultrasonography was the most commonly used diagnostic test. Hydronephrosis occurred in 54.4% of the cases, 81.1% of the stones were in the kidneys, and bilateral stones were associated to a family history of urolithiasis (p=0.030). Recurrence rate was 29.3% (most patients had a metabolic disorder). In 12.3%, the patients underwent lithotripsy, 24.5% were surgically treated (mainly pyelolithotomy), and only 7.6% had their stones analyzed (calcium oxalate was the main finding in the examined stones).

**Conclusions:**

The frequency of urolithiasis in these pediatric patients was similar to that reported by the literature. A metabolic evaluation is required and the composition of stones should be better evaluated.

## RESUMO

**Objetivo:**

Descrever a frequência, o perfil clínico e condutas adotadas em portadores de urolitíase no setor de nefropediatria do Hospital Federal dos Servidores do Estado na cidade do Rio de Janeiro.

**Métodos:**

Estudo retrospectivo dos prontuários de pacientes portadores de urolitíase, atendidos entre janeiro de 2012 e dezembro de 2014, com idade entre 1 mês e 18 anos. Variáveis estudadas: dados demográficos, antropométricos, quadro clínico, história familiar de urolitíase, infecção urinária e uso de medicamentos litogênicos, condutas diagnósticas, anomalias associadas, distúrbios metabólicos, terapêutica e recorrências.

**Resultados:**

A frequência de urolitíase no período foi de 13,6%, e as características mais frequentes foram sexo masculino, cor da pele branca, eutrofia, idade entre 5 e 10 anos, história familiar de urolitíase, infecção urinária prévia e eliminação espontânea do cálculo. Dor abdominal, em flanco e hematúria macroscópica foram as queixas mais comuns. Distúrbios metabólicos mais frequentes: hipercalciúria, hiperuricosúria e hipocitratúria. A hipocitratúria foi associada à história de infecção urinária prévia (p=0,004). A ultrassonografia de abdome ou aparelho urinário foi o exame mais utilizado para diagnóstico. Hidronefrose ocorreu em 54,4% dos casos, 81,1% dos cálculos estavam nos rins e os bilaterais eram associados com história familiar de urolitíase (p=0,030). Houve recidiva em 29,3% dos casos (maior parte com distúrbio metabólico); 12,3% submeteram-se à litotripsia; 24,5%, à cirurgia, principalmente pielolitotomia; e apenas 7,6% dos pacientes tiveram cálculos analisados (mais frequente: oxalato de cálcio).

**Conclusões:**

A frequência de urolitíase nessa população pediátrica foi próxima à da literatura. Os achados sugerem a necessidade de investigação metabólica mais ampla e a análise mais frequente dos cálculos.

## INTRODUCTION

Urolithiasis in childhood and adolescence has been increasingly diagnosed in the past three decades, in several countries of the world.^1^ The reason for such an increase is not clear, but is associated to climactic (global warming seems to predispose to urolithiasis, due to the reduced urine output and to insufficient water intake),^2^ and diet changes (foods rich in sodium, animal protein and carbohydrates, typical from industrialized countries, would favor the formation of calculi),^3^ genetic inheritance and, possibly, other environmental factors.^4^

The subjacent causes for urolithiasis for an expressive part of pediatric patients are metabolic disorders, infections and anomalies in the urinary tract.^5^ ^,^ ^6^ Urolithiasis is associated to an increased risk of chronic kidney disease, so early detection is important for these patients.^7^

A systematic review about this pathology, published by López and Hoppe, indicates there are few pediatric epidemiological studies in Latin America.^1^ The determination of the characteristics of these patients is essential to identify the possible predisposing factors and change them, whenever possible, thus reducing the morbidity of the disease and the costs associated with its recurrence.^8^ There are few data on the sample of urolithiasis in children in Brazil.

In this context, this study aimed at assessing the frequency and the clinical profile of the patients characterized as having urolithiasis, regularly followed-up in a three-year period, in the outpatient clinic of nephropediatrics of Hospital Federal dos Servidores do Estado (HFSE), Rio de Janeiro, Brazil.

## METHOD

By consulting the Statistics and File sector of HFSE, we found all patients followed-up in the nephropediatrics outpatient clinic of the hospital, from January, 2012, to December, 2014, and selected the charts with the urolithiasis diagnosis. The patients included in the study continued their regular follow-up at the hospital after the analysis.

The study was approved by the Human Research Ethics Committee at HFSE and Universidade Federal Fluminense (UFF). The patients of the selected sample came from Rio de Janeiro and Baixada Fluminense, where they were submitted to high temperatures for most of the year, which is a predisposing factor for urolithiasis. 

The inclusion criteria were:

1. Age between 1 month and 18 years;
2. Confirmation of clinical diagnosis by at least one radiological exam: simple abdomen x-ray, abdominal or urinary tract ultrasound (which can identify calculi ≥5 mm) and abdominal helical computed tomography without contrast (which identifies calculi of up to 1 mm - however, it should only be indicated when the previous modality is not clear and the symptoms persist, due to the risk of radiation);
3. Being assisted in the nephropediatrics outpatient clinic of HFSE in the aforementioned period.

Patients with suggestive clinical picture, however, without radiological confirmation, were excluded.

For being a reference center without an emergency unit, none of the assisted patients was assessed and treated in the acute phase, and some examinations were carried out outside the hospital.

In the study period, the routine of metabolic evaluation of the service included a 24-hour urine test, or in a single morning sample for patients with difficulties in collection (two samples), for the dosage of calcium, uric acid, citrate, oxalate, magnesium, cystine and phosphate, besides the serum dose of fasting glucose test. The normal values of the factors excreted in the 24-hour urine and in a single test, corrected with creatinine, used in the classification of the detected metabolic disorders, are in Table 1.^8^

**Table 1:** Normal values of excretion of components in the 24-hour urine and in the single sample, corrected by creatinine, in pediatric patients.

	24-h urine	Single sample (relation with creatinine)			Tubular reabsorption by the GFR
Creatinine	Up to 3 years: 6-22 mg/kg/day				
	>3 years: 12-30 mg/kg/day				
Calcium	<4 mg/kg/day (0.10 mmol/kg/day)	Age	mg/mg	mmol/mmol	
		0-6 m	<0.80	<2.24	
		6-12 m	<0.60	<1.68	
		1-2 years	<0.40	<1.12	
		2-18 years	<0.21	<0.56	
Citrate	≥400 mg/g creatinine/day	≥0.28 (mmol/L/mmol/L)			
Calcium/citrate	<0.33	<0.33			
Uric acid	<815 mg/1.73 m^2^/BS	<0.65			
Cystine	<60 mg/1.73 m^2^/BS	<0.02 mg/mg; <0.01 mmol/mmol			
Magnesium	>88 mg/1.73 m^2^/BS				
Oxalate	<50 mg/1.73 m^2^/BS or <0.49 mmol/1.73 m^2^/BS	Age		mg/mg	
		0-6 m		<0.30	
		7 m-4 years		<0.15	
		>4 years		<0.10	
Phosphate					>2.8 and <4.4 mg/dL

GFR: glomerular filtratrion rate; BS: body surface. Source: Penido and Tavares.^8^

All data were collected based on information in from the charts. The variables analyzed were: demographic (sex, skin color declared by the person responsible for the patient, age at the onset of the clinical picture and diagnosis, with stratification by age group in younger than 5 years, aged between 5 and 10 years, and between 10 and 18 years); anthropometric (weight, height, and body mass index (BMI)/age, measured in the first appointment); signs and symptoms referred in the first appointment; positive family history for lithiasis and level of kinship; history of previous urinary infection; history of use of lithogenic drugs before the diagnosis and identification of type; radiological examination carried out with information about the location of the calculi, identification of the anomaly and presence or absence of bilateral or multiple lithiasis; presence of metabolic disorder presenting risk for urolithiasis; history of spontaneous elimination of calculi; drugs used; performance of extracorporeal lithotripsy (ECLT); need for surgery and identification of type; recurrence and interval in between episodes; analysis of calculi and identification of type.

The statistical analysis required the use of software Stata, version 8.0 (StataCorp LP). The comparison between the frequencies was carried out using the chi-squared and Fisher’s exact test, and the Pearson correlation was used to investigate the correlation between the continuous variables. The statistical significance level adopted was p<0.05.

## RESULTS

In the analyzed period, 969 patients were assisted at the nephrology sector of the studied hospital. Of these, 780 charts were available for data collection; 106 met the inclusion criteria, resulting in frequency, in the period, of 13.6%. The demographic characteristics of the sample are described in Table 2.

**Table 2:** Demographic characteristics of the patients being followed-up at Hospital Federal dos Servidores do Estado, between January 2012 and December 2014.

	n	%
Gender		
Female	52	49.1
Male	54	50.9
Skin color		
White	67	63.2
Brown	30	28.3
Yellow	1	0.9
Black	8	7.6
Age at the onset of symptoms^a^ (years)		
<5	17	16.0
≥5 to ≤10	54	50.9
>10 to ≤18	35	33.0
Age of diagnosis^b^ (years)		
<5	8	7.5
≥5 to ≤10	52	49.1
>10 to ≤18	46	43.4

Total of the sample: n=106; ^a^mean 8.9±3.8; ^b^mean 9.9±3.6.

The age at the onset of symptoms and that at the time of diagnosis were positively correlated, indicating correspondence between these values (Perason 0.8619; p<0.001). Age at the time of diagnosis ranged from 3 to 18 years (Figure 1).

**Graph 1:**
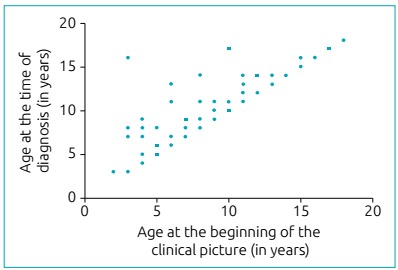
Correlation between age at the beginning of the clinical picture and at the time of diagnosis (r=0.8619; p<0.001).

The weight/age indicator, used in 54 patients (according to orientation from the World Health Organization - WHO, it is only applied until the age of 10 years), revealed that 45 (83.2%) had adequate weight in all age groups. The height/age indicator, used in 101 patients - 5 patients did not have that record -, showed that 97 (96.0%) presented adequate height in all age groups. The BMI/age indicator, also used in 101 patients - 5 patients did not have record of height, thus preventing the BMI calculation -, revealed that 66 (65.3%) were eutrophic. The nutritional diagnosis of overweight or obesity was observed out in 30 (29.7%) patients.

Figure 2 shows the signs and symptoms referred in the first episode of lithiasis, and most patients presented more than one sign or symptom, without differences between the age groups. In 69 (65%) patients, there was report of previous urinary infection, and 65 (61.3%) had positive family history for urolithiasis, being 55.7% with first-degree kinship. Five patients used lithogenic drugs before diagnosis (topiramate in three cases, and methotrexate in two cases).

**Graph 2:**
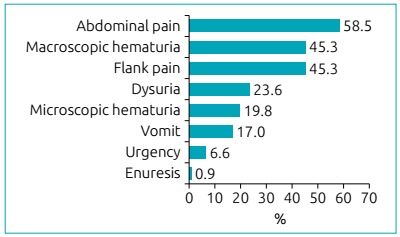
Frequency of signs and symptoms referred by the patients.

The most used radiological examination was abdominal or urinary tract ultrasound, in 101 (95.3%) cases, whereas abdominal computed tomography was carried out in 44 (41.5%). In 40.6% of the cases, more than one test was performed. Structural or functional anomalies were found in 46 (43.4%) patients, and hydronephrosis was the most frequent one (54.4%). Multiple calculi were found in 33 (31.1%) cases, and, bilaterally, in 24 (22.6%). Bilateral calculi were more common among patients with positive family history of urolithiasis (Fisher test; p=0.030). The most frequent location of the calculi were the kidneys - 86 (81.1%) cases.

The metabolic disorders detected are described in Table 3. Twelve (11.3%) patients did not undergo examinations, and, in 19.1% of the cases, no disorder was found. Hypocitraturia was more common in the age group of 5 to 10 years (Fisher test; p=0.001), and was strongly associated with previous urinary infection (Fisher test; p=0.004).

**Table 3:** Frequency of metabolic disorders found in patients being followed at Hospital Federal dos Servidores do Estado, between January 2012 and December 2014.

Metabolic disorder	n	%
Hypercalciuria	36	34.0
Hyperuricosuria	34	32.6
Hypocitraturia	31	29.3
Hypomagnesiuria	13	12.3
Hyperoxaluria	3	2.8
Cystinuria	2	1.9
Hyperphosphaturia	2	1.9
Hyperglycemia	2	1.9

Total of the sample: n=94.

Table 4 shows the drugs used after the metabolic investigation of the patients. As to evolution, 67 (63.2%) cases showed the spontaneous elimination of the calculi, mostly in the age group between 5 and 10 years. The lowest elimination rate was among the ones younger than 5 years. Thirteen (12.3%) patients were submitted to ECLT, however, only 7 had a positive resolution. Twenty-six (24.5%) patients were submitted to surgery, and pyelolithotomy was the most common one (57.7%); the most affected age group was between 10 and 18 years.

**Table 4:** Frequency of the drugs used after the metabolic investigation by the patients being followed at Hospital Federal dos Servidores do Estado, between January 2012 and December 2014.

	n	%
Potassium citrate	54	50.9
Thiazide diuretics	22	20.8
Magnesium hydroxide	9	8.5

Total of the sample: n=106.

Recurrence was registered in 31 (29.3%) patients, and the interval in between episodes, in most cases - 74,2% -, was 12 to 24 months. In 96.8% of the patients with recurrence, some sort of metabolic disorder was detected. Only 8 (7.6%) patients had their calculi analyzed, and the most frequent one was calcium oxalate (50%).

## DISCUSSION

Penido and Tavares, in 2015, stated that the true frequency of urolithiasis in children remains unknown due to the multiplicity of pathogenetic factors, the lack of specificity of the clinical picture at this age group and the lack of studies with appropriate scientific design.^9^ On the other hand, Schor and Heilberg estimated that the frequency of urolithiasis in Brazil was 10%.^10^ In this study, the frequency observed in the period was 13.6%. The possible causes of this higher frequency in relation to that suggested by Schor and Heilberg may be the high temperature of the city, and because it regarded a reference hospital.^10^ The knowledge of this frequency and its predisposing factors leads the care team to consider the growing importance of the theme, and the need for family participation in patient care.

Publications in developing countries registered a variable proportion between the male and female genders, of 1.2:1 until 4:1.^4^ In this study, the proportion was 1.03:1, which is compatible with North-American series, according to Penido et al.^11^ North-American series suggest that the mean age at the time of diagnosis is 11.3 to 13.2 years.^11^ In this study, the mean was 9.9±3.6. It is possible that, once again, the high temperature of the city may have contributed with this result. A recent Brazilian publication found mean age of the patients in the beginning of the symptoms to be close to this study.^4^ Sas et al.^3^ stated there is lower risk of development of urolithiasis in people aged less than 5 years, unlike adolescents, which was also confirmed in this study. Shoag et al.^7^ claimed urolithiasis is more common among students and adolescents, as well as in white patients, and this finding is similar to this study.

The delayed diagnosis in children aged less than 5 years can cause risks to kidney function.^3^ This result was found in this sample, suggesting that more attention should be addressed to this age group due to the severity of a late diagnosis. The correlation between age in the onset of symptoms and that at the time of diagnosis was strongly positive, suggesting that, at an individual level, these ages were dependent, that is, the symptoms effectively corresponded to urolithiasis.

Bandari et al.^12^ detected major hypocitraturia, hypomagnesiuria, and hypercalciuria in children with overweight or obesity. It is known that citrate and magnesium are natural inhibitors of urinary crystallization, and calcium is the most common component in urinary calculi in pediatric patients.^9^ There is still no relationship established between pediatric urolithiasis and overweight or obesity, unlike what happens for adult patients whose BMI higher than the 85 percentile is considered as a risk factor for urolithiasis. On the other hand, Kim et al.^13^ and Kieran et al.^14^ concluded that high BMI is not associated with urolithiasis in children, which makes it controversial. As to the BMI/age indicator in this study, 29.7%, that is, almost one third of the patients, were above the 85 percentile. It is possible that dietary habits can explain such an association.

Amancio et al.^4^ found the same symptoms as being the most frequent ones. According to these authors, the report of urinary infection takes place in up to 70% of the cases, and it can be a cause or a consequence of urolithiasis. A high percentage of urinary infections (65.1%) was detected in the study sample, showing the importance of studying lithiasis in patients with urinary tract infection.

Family history of urolithiasis is detected in a variable frequency, occurring, according to Amancio et al.^4^, in 85% of the patients, and, according to Hoppe and Kemper,^15^ in 40%. Sharma and Filler^2^ state that first-degree relative have 2 to 16 times more chances of developing the disease. In this sample, 61.3% of the analyzed patients reported positive family history, and 55.7% had first-degree relatives with the disease. Such findings reinforce the importance of obtaining this information in the anamnesis.

Almost 90% of the calculi can be diagnosed by ultrasound, carried out by an experienced professional, according to a recent review by Morrison et al.^16^ According to these authors, ultrasound to diagnose urolithiasis in children has sensitivity of 76% and specificity of 100%, in comparison to tomography, whose sensitivity is 98%, especially for calculi located in the ureter.^16^ These authors also suggest that tomography be reserved only for the preoperative investigation and, in dubious cases, with protocols of low doses of radiation.^16^ In this study, abdominal or urinary tract ultrasound were the most used radiological examinations for diagnostic confirmation - test recommended by the literature for not exposing the patient to radiation, and because the size of non-visualized calculi is clinically insignificant.^16^ On the other hand, 63% of the patients studied by Tasian and Copelovitch underwent computed tomography,^17^ whereas, in this study, 41.5% of the patients did so, showing the frequent use of this examination for diagnosis. It is possible that the continuous education of the care team can reduce such a percentage.

The detection of bilateral calculi associated with positive family history is a characteristic of hereditary and monogenic lithiasis, so early diagnosis is important to prevent severe kidney damage,^18^ and this association is found in this study. Alpay et al.^19^ observed the most frequent location of the calculi was the kidneys, which is similar to this study.

According to the literature, metabolic disorders occur in 33 to 93% of the cases.^4^ ^,^ ^11^ In this study, at least one change was found in 80.9% of the cases, which draws the attention to the need for this investigation. According to Copelovitch,^20^ hypercalciuria occurs in 30 to 50% of the cases; in this study, this was the most common change (34.0%). Iranian studies^21^ and from Amancio et al.^4^ pointed to hyperuricosuria as a frequent metabolic change, similarly to the findings in this study (32.6%), which possibly is related to dietary aspects. Rellum et al.^22^ highlight the high frequency of hypocitraturia, which occurred in 29.3% of the patients in this sample. Kovacevic et al.^23^ pointed out to the importance of the association between previous urinary infection and hypocitraturia, and this result was confirmed in this study. In the study by Penido,^24^ 24.3% of the cases showed no metabolic change, only the reduction in urine volume (normal value: ≥1 ­mL/­kg/h). These findings suggest that the dietary investigation and the urinary metabolic analysis are essential to the follow-up of these patients. It is known that the intake of liquids rich in fructose and sodium, added to the low consumption of water, result in metabolic changes that enable the formation of urinary calculi.^25^

According to the recommendation of urolithiasis of 2015, from the European Association of Urology^26^ and the review performed by Copelovitch,^20^ urinary pH, urine Na/K ratio and urinary oversaturation of calcium oxalate and uric acid are important items in the metabolic evaluation of patients, however, these items were not reported in the studied sample.

Penido and Tavares^8^ found the spontaneous elimination of the calculi to be between 60 and 70% in up to 6 weeks. In this study, this rate was 63.2%. The interesting aspect was, as it was for Sas et al.,^3^ the lowest rate of elimination in children aged less than 5 years. This fact, associated with the delayed diagnosis in this age group, could interfere in the prognosis of these patients. Amancio et al.^4^ reported a percentage of patients submitted to ECLT and to surgery close to the percentage found in this study, revealing therapeutic practices in accordance with the literature. It is worth to mention that ECLT is a safe treatment, with minimal complications, mainly indicated for calculi in the renal pelvis and proximal ureter.^8^ ^,^ ^9^

The most used pharmacological treatment after the metabolic investigation, according to Chu et al.^27^ and Penido and Tavares,^8^ was the same observed in this study (potassium citrate and thiazide diuretics), which is important for the reduction of the recurrence frequency. It is worth to mention that the increasing water intake, leading to increased urine volume, must be adopted regardless of the pharmacological approach.

 The risk of recurrence in pediatric patients is high, ranging from 19 to 34%.^27^ In the analyzed sample, the rate was 29.3%. Another relevant fact is the association of recurrence with metabolic disorders,^27^ observed in 96.8% of the patients with recurrence in this study. This fact shows the need for a more detailed metabolic analysis and for an appropriate pharmacological intervention to prevent recurrence. The calcium/creatinine and calcium/citrate urinary relations (Table 1) were considered by DeFoor et al.^28^ as excellent indicators of this risk; however, they were not registered in the records.

Amancio et al.^4^ reported the low frequency of the analysis of the calculi, which was very similar to this study. In most cases, the calculi was expelled in the patients’ houses, and neither them nor their relatives had been informed about the importance of collecting the item for analysis. It is worth to remember that it is not always possible to predict the composition of the stone through urine composition.^15^ The type most frequently reported in the literature (40 to 65%) is the calcium oxalate,^20^ and this is the finding in the studied sample.

A few authors have explored the frequency of use of potentially lithogenic drugs in children. Hoffmeister et al.^29^ reported that 4.7% of the patients who used methotrexate presented with urolithiasis, whereas Mahmoud et al.^30^ referred that 5.2% of the patients who used topiramate also presented with urolithiasis. In the sample of this study, the frequency of use of these drugs was 4.7%. The study by Amancio et al.,^4^ which refers to the national sample, did not explore that aspect. It is extremely importante that patients using these drugs be monitored and oriented, aiming at prevention and early diagnosis.

The main study limitations were: retrospective design with absence of homogeneous data in the records, small number of stones analyzed, and absence of data for a complete metabolic investigation.

The conclusion is that the frequency of urolithiasis in the nephropediatrics outpatient clinic at HFSE was close to that estimated by national authors; the main characteristics of the studied sample are in accordance with the national and international literature. Children aged less than 5 years presented lower rates of calculi elimination, and may represent a group with more difficulties in diagnosis and severity. The metabolic analysis was not carried out in all children. Given the importance and association with recurrence rates, besides the need for specific treatment, it is essential that the analysis of the calculi and the metabolic study of these patients be carried out by the assisting team. It is necessary to create a protocol for a uniform follow-up of these patients.

## References

[1] López M, Hoppe B (2010). History, epidemiology and regional diversities of
urolithiais. Pediatr Nephrol.

[2] Sharma AP, Filler G (2010). Epidemiology of pediatric urolithiasis. Indian J Urol.

[3] Sas DJ, Becton LJ, Tutman J, Lindsay LA, Wahlquist AH (2016). Clinical, demographic and laboratory characteristics of children
with nephrolithiasis. Urolithiasis.

[4] Amancio L, Fedrizzi M, Bresolin NL, Penido MG (2016). Pediatric urolithiasis: experience at a tertiary care pediatric
hospital. J Bras Nefrol.

[5] Sas DJ (2011). An update on the changing epidemiology and metabolic risk factors
in pediatric kidney stone disease. Clin J Am Soc Nephrol.

[6] Hope B, Leumann E, Milliner DS, Geary DF, Schaefer F (2008). Urolithiasis and nephrocalcinosis in childhood. Comprehensive pediatric nephrology.

[7] Shoag J, Tasian GE, Goldfarb DS, Eisner BH (2015). The new epidemiology of nephrolithiasis. Adv Chronic Kidney Dis.

[8] Penido MG, Tavares MS (2015). Pediatric primary urolithiasis: symptoms, medical management and
prevention strategies. World J Nephrol.

[9] Penido MG, Tavares MS (2015). Nefrologia Pediátrica: manual prático.

[10] Schor N, Heilberg IP (2015). Litíase renal: manual prático.

[11] Penido MG, Srivastava T, Alon US (2013). Pediatric primary urolithiasis: 12-year experience at a
Midwestern Children's Hospital. J Urol.

[12] Bandari J, Dangle PP, Lyon TD, Lee A, Schneck FX, Cannon GM (2016). 24-hour urinary parameters in overweight and obese children with
urolithiasis. J Urol.

[13] Kim SS, Luan X, Canning DA, Landis JR, Keren R (2011). Association between body mass index and urolithiasis in
children. J Urol.

[14] Kieran K, Giel DW, Morris BJ, Wan JY, Tidwell CD, Giem A (2010). Pediatric urolithiasis - does body mass index influence stone
presentation and treatment?. J Urol.

[15] Hoppe B, Kemper MJ (2010). Diagnostic examination of the child with urolithiasis or
nephrocalcinosis. Pediatr Nephrol.

[16] Morrison JC, Kawal T, Batavia JP, Srinivasan AK (2017). Use of ultrasound in pediatric renal stone diagnosis and
surgery. Curr Urol Rep.

[17] Tasian GE, Copelovitch L (2014). Evaluation and medical management of kidney stones in
children. J Urol.

[18] Ferraro PM, D'Adessi A, Gambaro G (2013). When to suspect a genetic disorder in a patient with renal
stones, and why. Nephrol Dial Transplant.

[19] Alpay H, Ozen A, Gokce I, Biyikli N (2009). Clinical and metabolic features in children. Pediatr Nephrol.

[20] Copelovitch L (2012). Urolithiasis in children medical approach. Pediatr Clin North Am.

[21] Fahimi D, Zoham MH, Sheikh M, Salabati M, Ghazanfani A, Fironzi M (2016). A comparison between clinical and metabolic features of renal
calyceal microlithiasis and overt urolithiasis in different pediatric age
groups. Urol Int.

[22] Rellum DM, Feitz WF, Herwaarden AE, Schrender MF (2014). Pediatric urolithiasis in a non-endemic country: A single center
experience from The Netherlands. J Pediatr Urol.

[23] Kovacevic L, Wolfe-Christensen C, Edwards L, Sadaps M, Lakshmanan Y (2012). From hypercalciuria to hypocitraturia - a shifting trend in
pediatric urolithiasis?. J Urol.

[24] Penido MG, Tavares MS, Guimarães MM, Srivastava T, Alon US (2014). American and brazilian children with primary urolithiasis:
similarities and disparities. Global Pediatr Health.

[25] Roudakova K, Monga M (2014). The evolving epidemiology of stone disease. Indian J Urol.

[26] Türk C, Knoll T, Petrik A, Sarica K, Skolarikos A, Straub M (2015). Guidelines on urolithiasis.

[27] Chu DI, Tasian GE, Copelovitch L (2016). Pediatric kidney stones - avoidance and treatment. Curr Treat Options Ped.

[28] DeFoor W, Minevich E, Jackson E, Reddy P, Clark C, Asplin J (2008). Urinary Metabolic Evaluations in solitary and recurrent stone
forming children. J Urol.

[29] Hoffmaister PA, Storer BE, Baker KS, Hingorani SR (2014). Nephrolithiasis in pediatric hematopoietic cell transplantation
with up to 40 years of follow-up. Pediatr Blood Cancer.

[30] Mahmoud AA, Rizk T, El-Bakri NK, Riaz M, Dannawi S, Al Tannir M (2011). Incidence of kidney stones with topiramate treatment in pediatric
patients. Epilepsia.

